# The Golden Spiral Flap: A New Flap Design that Allows for Closure of Larger Wounds under Reduced Tension – How Studying Nature’s Own Design Led to the Development of a New Surgical Technique

**DOI:** 10.3389/fsurg.2016.00063

**Published:** 2016-11-16

**Authors:** Sharad P. Paul

**Affiliations:** ^1^Department of Skin Cancer, School of Medicine, University of Queensland, Brisbane, QLD, Australia; ^2^Faculty of Surgery, University of Auckland, Auckland, New Zealand; ^3^Auckland University of Technology, Auckland, New Zealand

**Keywords:** skin, skin neoplasms, dermatology, scalp, surgical flaps, surgical procedures, operative, plastic surgery

## Abstract

This paper details the study of biodynamic excisional skin tension lines on the scalp and the development of a new flap technique for closure of scalp wounds. Recently, a study by this author, on pigskin, replicated whorls by placing tissue under rapid stretch using saline tissue expanders, by recreating rapid dermo-epidermal shear of skin – thereby concluding that the golden spiral pattern is nature’s own pattern for rapid expansion. Given the relationship between tissue expansion and stretch has been shown to cause deformation gradients that have both elastic and growth factors, the author set out to test the hypothesis that a golden spiral pattern therefore would be more efficient at closing wounds under less tension when compared with standard semicircular rotational flap patterns. The author conducted a series of experiments, both on pigskin (to first confirm the hypothesis, using a recently developed computerized tensiometer) and later a clinical study. This paper presents a new random pivotal flap technique for skin closures on the head and neck: the golden spiral flap. Biomechanics, planning, and advantages of this new flap are described in this paper.

## Introduction

Achieving tension-free, primary wound closure is the goal in any reconstructive surgical procedure, and to this end, surgeons have tried undermining wounds or imbrication to preserve dermal perfusion while still reducing tension at the wound edge (1). Others wondered if planning incisions in particular directions may also hold the key – and beginning with Langer’s description (2) of “cleavage lines” in 1861, many authors have studied and mapped out cutaneous “skin tension” and wrinkle lines and adopted them for surgery. While understanding that the tensile strength of a wound is important for wound closure (3), there has been poor scientific correlation between cutaneous wrinkle lines and wound tension. A team led by this author has used computational analyses to understand biodynamic excisional skin tension (BEST) lines, i.e., the best lines of wound closure, after tissue has been removed by excision of skin lesions (4). The study began on skin of the scalp for two reasons – it is relatively untethered to underlying tissues, and scalp whorls that appear to confirm to nature’s golden spiral pattern are unique to human beings – and were thought to occur due to the rapid expansion of the brain during intrauterine life. While golden spiral patterns are evident in nature, there were considered mysterious until a recent study on skin has suggested that this may indeed be nature’s pattern for rapid expansion (5). Such studies have recently demonstrated that when expansion of tissues was not rapid, whorl patterns did not result and that the golden spiral pattern may be useful as an intra-operative pattern for stretch. It has also been established that biomechanically, skin behaves more like an elastic solid – a shell draped on a continuum foundation (6) This continuum mechanics approach has show to be of great use in understanding skin stretch, skin tension, and tissue expansion, especially on sites such as the scalp (7) Also, the golden spiral pattern has previously been shown to be a pattern for rapid expansion of organic tissues (5) – and recent biomechanical studies on the scalp and the resultant mechanical theories all point to a continuum framework for finite growth (8, 9). Therefore, studies suggest that, especially on the scalp, expansion and tension are interrelated, and the relationship between this stretch and expansion works like this: when skin is deformed either due to scalp expansion or stretch (as in the case of pivotal flaps), the deformation gradient has an elastic and a growth part (10).

The hypothesis that resulted as a corollary of this finding that a golden spiral pattern resulted from rapid expansion suggested that utilizing nature’s own grand design, the golden spiral, could also be a method of stretching skin more efficiently during surgery – and this reduced wound tension would result in easier wound closure, improved growth factors, and better wound healing. It was an experimental testing of this hypothesis, both on pigskin and a clinical study – that has led to a new surgical method for scalp closure that is a major improvement on previous rotation flap techniques.

Semicircular geometric incisions have been often utilized as a method of closing scalp skin defects (11). Single, double, and triple pedicled rotational flaps have been described previously to close scalp skin defects by pivoting skin about a base and sliding the semicircular skin flaps into place (12). In general, these are hemicircular pivotal flaps, and closure of a defect is performed by gently moving skin about a pivot, along the perimeter a circle. A series of experiments on rotational scalp tension by Larrabee concluded that tension was concentrated at 90° and 135° – in other words, extending the semicircle of the flap beyond 135° did not allow for easier closure (13).

While whorl and golden spiral patterns abound in nature in plants and space, on human scalp skin, these whorls are significant for two reasons – every human has a scalp whorl, and these patterns are unique and different, even in twins (14). The mechanical theory of scalp whorl formation suggests that during the 10th–12th week of fetal life, the brain expansion is so rapid that it creates a shearing force between the two layers of skin, at the dermo-epidermal junction, which in turn causes the hair follicles to curve – resulting in the golden spiral pattern formation (15). This remained a theory only, until recently, when a study by the author on pigskin replicated whorls by placing tissue under rapid stretch using saline tissue expanders and recreating rapid dermo-epidermal shear of skin (5).

To test the hypothesis that a golden spiral pattern therefore would be more efficient at stretching skin to cover a defect when compared with standard semicircular patterns, the author conducted a series of pigskin experiments. As the pigskin experiments demonstrated a significant reduction in tension using a golden spiral flap (as opposed to a traditional rotational flap), this was then validated by a clinical study on large scalp defects. As noted earlier, biomechanical studies undertaken on traditional hemicircular rotational flaps did not show any reduction in tension beyond 135° – and this led to the use of a back-cut or a hatchet flap to aid closure. This hatchet technique, first described by Emmett (16) is a triangular local rotation flap, with a degree of advancement and with a back-cut at the base of the flap, through which it derives its vascular supply. As is the case with rotational flaps, it can be planned with one or two pedicles. During preliminary testing, the author experimented with both rotation flaps with back-cuts and hatchet flaps – and found that they did not reduce tension greater than a standard hemispherical rotation flap, and therefore, the decision was made to compare the standard rotation flap with the golden spiral flap design, in keeping with the hypothesis being tested.

## Materials and Methods

**Figure 1:**
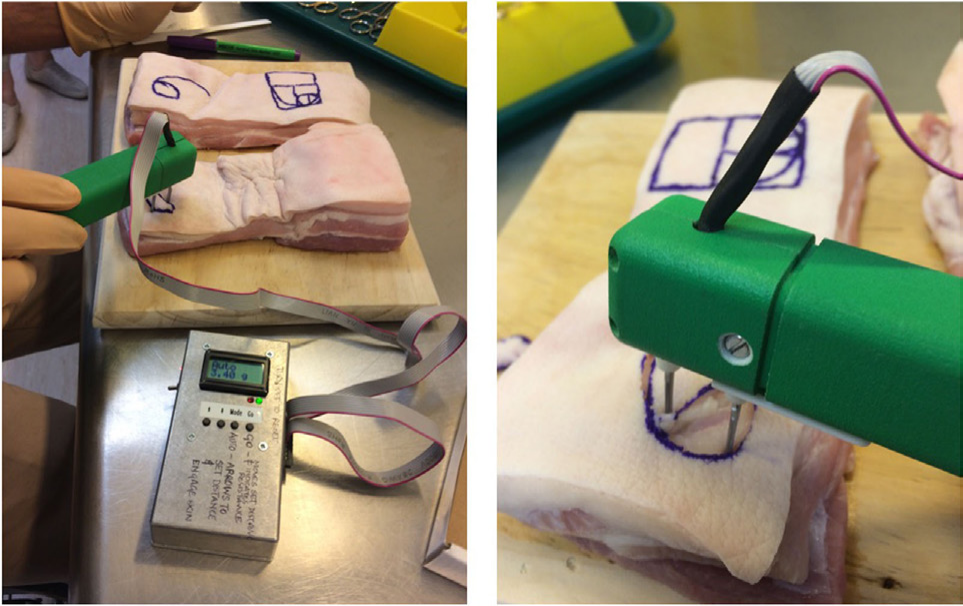
**Flap design tension comparisons using tensiometer on pigskin**.

Pigskin was used as this has been established as surgically comparable to human skin (17). Pigskin obtained from 20 different animals was tested as part of this study. The pigskin, freshly slaughtered, was obtained from an abattoir (supplied by FreshPork Northern, with the animals sourced from Timaru, New Zealand); and 10 pig bellies and 10 pig heads were used, so the results could be compared on different anatomical regions. The 1- and 2-cm circular full-thickness defects (cut-outs of full-thickness skin) were created, and flaps were created to close these defects – either using the traditional semicircular rotation flap design or the golden spiral design. The pigskin blocks were glued to the underlying boards using contact adhesive glue to prevent any underlying motion that could affect measurement (3M 30NF Green Fastbond Contact Adhesive, manufactured by 3M, St. Paul, MN, USA). Tension was measured at the point of maximum tension of the pivotal flaps, using a computerized digital tensiometer (Figure 1) that has been used to study skin biodynamics, determine the biomechanical differences between “tension” and “relaxation” skin lines, and demonstrate the best lines of closure, with least tension, after skin excisions (4). This tensiometer was especially developed by the author’s team to study biomechanics of skin – it consists of four main elements: a linear actuator, a force sensor, signal conditioning hardware, and embedded software. To take a tension measurement, the software starts by taking a calibration reading; this reading is known as the zero tension point. The algorithm to calculate the tension ultimately is the difference between two force measurements as described above; these force measurements are derived from a digital ADC reading (0–1023), which is converted to grams using the formula (*g* = ADC value × 0.25), and these were converted to Newton for ease of publication of results. This device is bidirectional, i.e., the user can measure inward and outward forces by flicking the switch to change the direction of measurement. This allows us to measure any inherent skin tension (pre-tension) and understand both skin tension and relaxation lines and details of this have been previously published (4).

The next stage of testing this hypothesis was a formal study on patients, after obtaining all the necessary ethics approvals [New Zealand Health and Disability Ethics Committee (HDEC Reference number 15/CEN/113); Australia: University of Queensland Institutional Human Ethics (Approval No. 2015001550)]. This study was carried out in accordance with the recommendations of ethics committees listed above, with written informed consent from all subjects. All subjects gave written informed consent in accordance with the Declaration of Helsinki. The results of the pigskin and clinical experiments are detailed below.

In each animal, identical (1 or 2 cm) defects were created and closing tension measured using the two different flap techniques  – standard rotation flap vs. the golden spiral flap. The results are found in Table 1. With regard to the statistical methodology – the *t* test we applied is a standard statistical methodology for comparing two different techniques or paired data. The null is that the two population means are identical, which is a reasonable and interpretable hypothesis – given each flap was tested on identical defects on the same animal.

**Table 1:** **Golden spiral and conventional rotational flap tension testing – force required to close the wound is recorded in Newton**.

No.	Site	Defect size (cm)	Rotation flap tension (N)	Golden spiral tension (N)	Variation %	Mean % reduction in tension
1	Belly	0.6	0.8	0.8	0	
2		0.7	1.0	1.0	0	
3		0.6	0.4	0.4	0	
4		0.7	0.8	0.8	0	
5		1	1.2	1.2	0	
6		2	1.6	1.2	−25	
7		2	2.0	1.3	−35	
8		2	2.1	1.2	−42.8	
9		2	1.5	1.2	−20	
10		2	2.7	2.0	−25.9	
11	Head	1	1.4	1.0	−28.5	
12		1	2.0	1.5	−25	
13		1	2.1	1.1	−47.6	
14		1	2.0	1.4	−30	
15		1	2.2	1.0	−54.5	
16		2	3.0	2.0	−33.3	
17		2	3.1	2.0	−35	
18		2	2.9	1.5	−48	
19		2	2.8	1.9	−32.1	
20		2	3.1	2.4	−22.5	
						25.2

We can assume that the trials are independent as there is no connection between different trials, given each comparison was on a different animal (each animal had measurements done once on each flap technique on similar body sites).

The assumption of normal distribution is standard in statistical methods, and there is no reason to suspect non-normality. The test had to be paired because the results were organized with each paired measurement representing a different animal. We did not assume equality of variance and used the Welch approximation for the *p*-value calculation. The detailed results are included in Section “Results.”

Planning and design of the golden spiral flap (Figure 3):

1. Draw two 1-unit squares.
2. Mark out an adjoining 2-unit square, followed by a 3-unit square and a 5-unit square.
3. The lesion to be excised (including clinical margins) should be located within the first three squares.
4. While mathematicians use a compass to mark golden spirals using this sequential square method, the author has developed a surgical short-cut to achieve the same: mark a diagonal ellipse across each square and discard the clockwise marking and connect the anti-clockwise markings to end up with a perfect golden spiral.
5. The flap usually closes in the fifth square and if needed can be continued beyond for larger defects (Video S1 in Supplementary Material).

This golden spiral design was compared with standard rotation flap design on freshly slaughtered pigs (no animals were slaughtered specifically for this study); fresh skin and pig heads were obtained from the abattoir to ensure the skin tensions were as close to live animals as possible. While a weakness of this study may be that we did not use living animals, this was reduced by the following factors that made the results statistically relevant and reproducible:

1. We used freshly slaughtered animals.
2. Each comparison was done on a different animal, i.e., each animal had both a rotation flap and a golden spiral flap done and tension measurements were compared.
3. As there was no relationship between the different animals, and each animal underwent the creation of similar 1- and 2-cm defects, the null hypothesis was that the two population means were identical, which the statistics team felt was a reasonable and interpretable hypothesis.

## Results

The recordings in Table 1 show the forces needed to close the wound using the two different flap techniques on pigskin.

The study showed that using the golden spiral flap allows us to close the wound under less tension, when compared to standard rotational flaps. The mean reduction in tension, in our study, was significant – at around 25%. A detailed statistical analysis was then undertaken by the mathematical sciences and statistics department that revealed the following: for both belly sites with a defect size ≥2 cm and for head sites with defect size of 1 or 2 cm; the golden spiral method resulted in significantly less tension than the rotation flap method [one-sided pooled variance Student’s *t*-test; *p* = 0.0027 (belly, 2 cm), *p* = 0.0043 (head, 1 cm), *p* = 0.00046 (head, 2 cm)].

**Figure 2:**
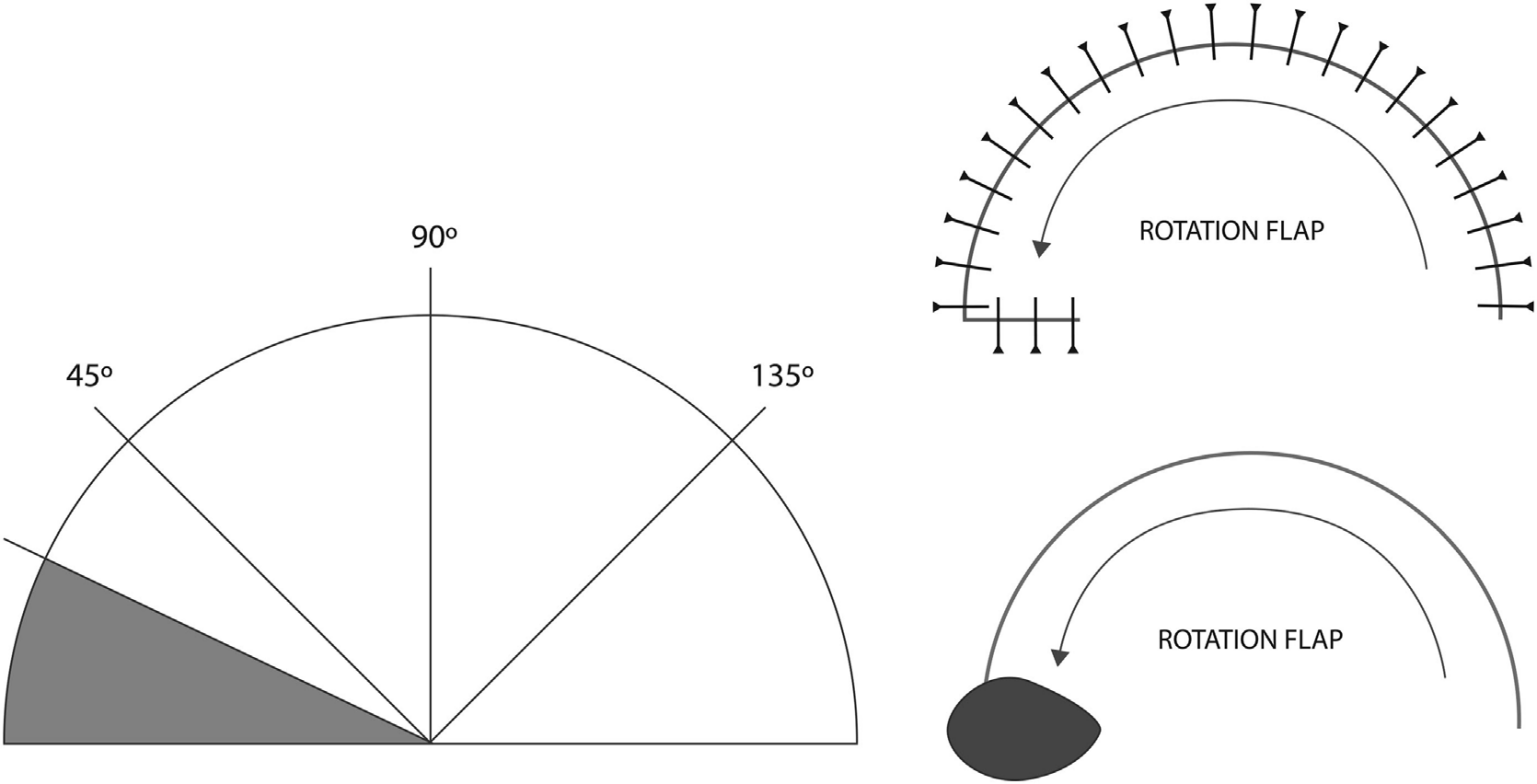
**Standard rotational flap design**.

**Figure 3:**
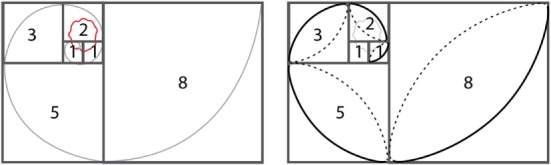
**Planning the golden spiral flap and design**.

There were some interesting findings – when the wound was very small, i.e., under 7 mm and the closing tension was low, there was not much difference between both the pivotal flap designs (Figures 2 and 3) – and surgically speaking, such wounds would be closed primarily without a flap anyway. Naturally, closing wounds on pig heads needed greater force than the force needed to close softer pig belly skin. However, as the defects became larger, the golden spiral flap proved much easier to close than a standard rotation flap, proving its superiority as a design capable of rapid stretch. During these pigskin experiments, it was noted that when conventional rotational flap designs were utilized, even the use of a back-cut reduced tension by less than 5% – and the golden spiral design facilitated much easier closure. This is because the golden spiral flap is, in essence, a logarithmic spiral flap with a growth factor of φ or the golden ratio (18) – what this means is that for every quarter turn the flap is rotated, it becomes progressively wider and therefore ends up closing wounds under less tension than a standard hemicircular flap.

**Figure 4:**
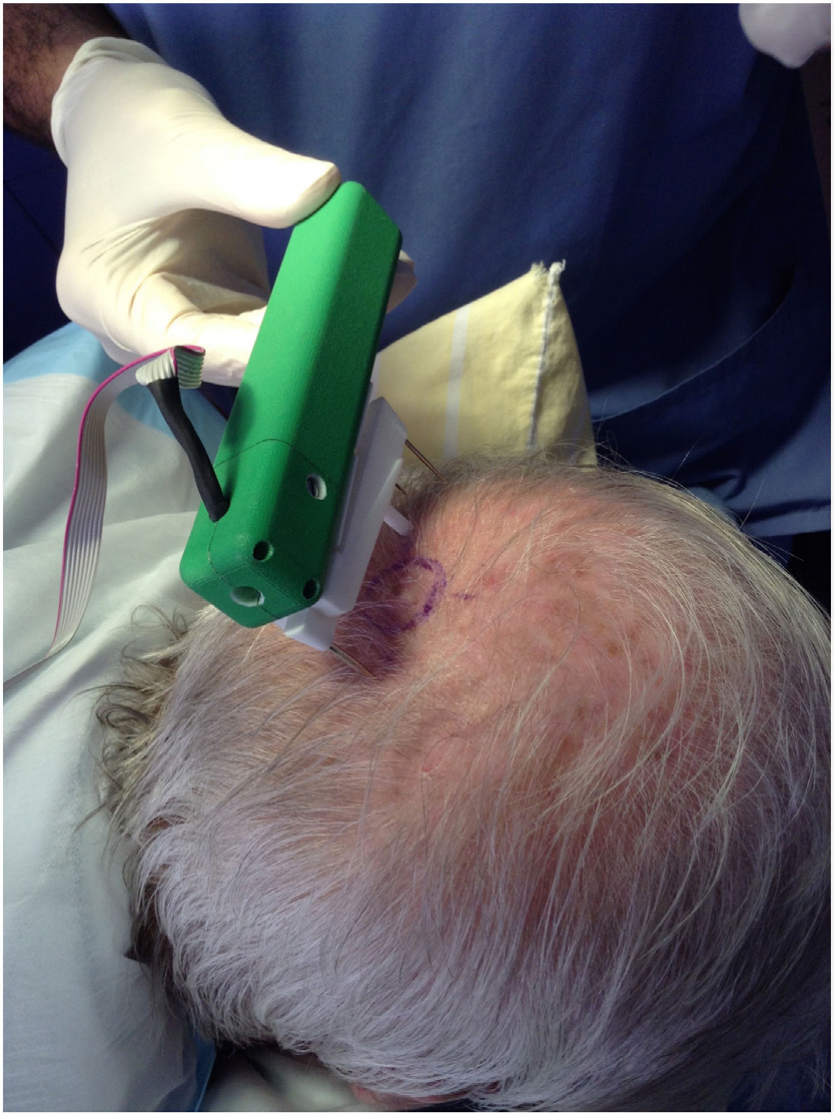
**Scalp tension measurement during surgery**.

**Figure 5:**
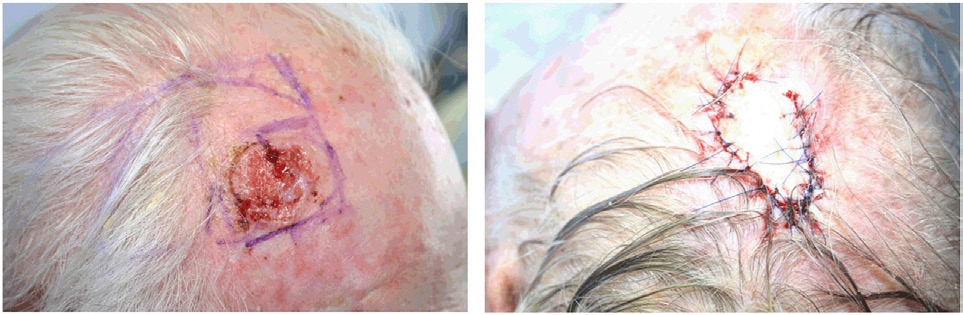
**Golden spiral flap (as each is one example of a flap design)**.

**Figure 6:**
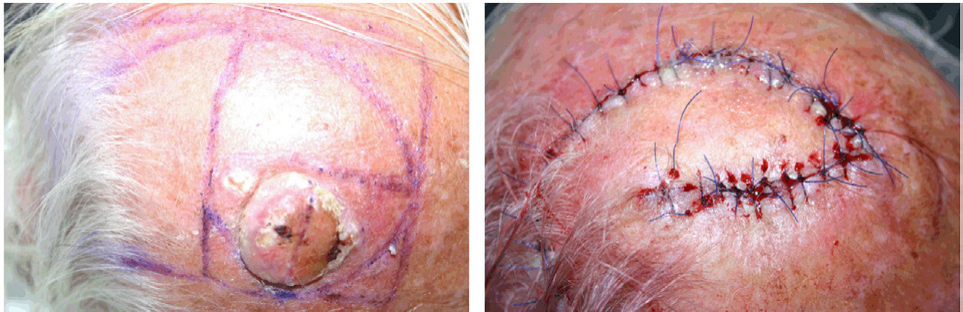
**Golden spiral flap (as each is one example of a flap design)**.

The results presented, in this initial series of 11 patients, confirm our earlier impressions after pigskin testing: a flap based on the golden spiral pattern, nature’s own design for expansion, achieves far superior results to a conventional semicircular rotational flap, i.e., it allows us to close defects under less tension than using a standard rotation flap. Table 2 provides further details regarding the size and pathology of lesions that were excised. Most patients in this study needed excisions for skin cancer. In previous studies done by the author on scalp skin cancers, forces required to close a wound varied from 0.5 to 4.6 N (4). The golden spiral flaps were planned and tension measured (Figure 4) as indicated in the clinical photographs – which show that even for 3- and 4-cm defects, closure was achieved relatively easily (Figures 5 and 6). Even though the defects ranged from 2 to 4 cm, closure was achieved without tension or the need for skin grafting. There were no wound complications (infection or dehiscence) in this series. The author notes that, on viewing the final result after wound closure, the golden spiral flap seems to have a narrower pedicle than was expected for this design, but this did not result in any flap necrosis or wound complications. Therefore, the author notes that while the design is similar to a rotational flap, the golden spiral flap is indeed a different kind of a random-pattern pivotal flap that is especially useful on the scalp, where random-pattern flaps are nourished by the large blood vessels located in the subcutaneous layer just superficial to the galea aponeurosis.

**Table 2:** **Pathology and size of lesions removed on the scalp**.

No.	Age (years)	Sex	Diagnosis on histology	Diameter of defect (cm)	Site
1	88	M	Ulcer/post-radiation necrosis	3	Scalp
2	50	F	BCC	3	Scalp
3	62	F	Trichilemmal cyst (proliferating)	4	Scalp
4	50	F	Basal cell cancer	3	Scalp
5	70	M	Squamous cell cancer	2	Scalp
6	68	M	Squamous cell cancer	4	Scalp
7	70	M	Basal cell cancer	2.5	Scalp
8	70	M	Basal cell cancer	2	Scalp
9	68	M	Basal cell cancer	3	Scalp
10	88	M	Squamous cell cancer	2.5	Scalp
11	65	M	Hypertrophic actinic keratosis	2	Scalp

## Discussion

The golden spiral flap presented here is a valuable new addition to a surgeon’s armamentarium when faced with large scalp defects, especially after skin cancer. Given the ease of using the sequential-Fibonacci-squares method to mark out the flap, it is adaptable to defects of various sizes easily. In general, the defect will lie within the first three squares, but given the nature of the design it is extremely adaptable. The dissection and undermining are done similar to other cutaneous flaps on the scalp, with dissection down to the loose areolar tissue. We have found that utilizing the golden spiral pattern on skin to be safe and reliable, with the added benefit of progressively lowered tension while closing larger defects. While the primary interest of this paper will be to a medical, dental, or veterinary audience, the author notes with interest that spiral patterns are increasingly studied in other media in recent times – Hakim and colleagues have studied spiral wave meander in excitable media and suggest that the dynamics of spiral waves can be reduced to a non-linear equation of motion for the wave tip that is asymptotically exact in a parameter range where the motion takes place around a large central core region (19). Other authors have studied the termination of pinned spirals on a defect by means of local stimuli (20). When looked at conceptually, these studies on spiral patterns are not unlike this experiment, and therefore, the utility of the golden spiral pattern in reducing wound closing tension is being presented to both the wider scientific community and, of course, a plastic surgical audience. The main application of this random-pattern pivotal golden spiral flap will be to facilitate closure of large scalp wounds where primary closure is not feasible. However, while the clinical study was performed only on the scalp, the detailed biomechanical analyses undertaken suggest that it is likely to be useful on other sites on the head and neck where one does not need a perforator-based flap.

## Author Contributions

SP was the sole author and conceptualized and conducted this study.

## Conflict of Interest Statement

The author declares that the research was conducted in the absence of any commercial or financial relationships that could be construed as a potential conflict of interest.

## Supplementary Material

Video S1 **Golden spiral flap animated video**.

## References

[1] Krishnan NM Brown BJ Davison SP Mauskar N Mino M Jordan MH Reducing wound tension with undermining or imbrication-do they work? Plast Reconstr Surg Glob Open (2016) 4(7):e799. 10.1097/GOX.0000000000000799 27536478 PMC4977127

[2] Langer K On the anatomy and physiology of the skin. Br J Plast Surg (1978) 31(1):3–8. 10.1016/0007-1226(78)90056-5 342028

[3] Milch RA Tensile strength of surgical wounds. J Surg Res (1965) 5:377–85. 10.1016/S0022-4804(65)80025-7 14321513

[4] Paul SP Matulich J Charlton N. A new skin tensiometer device: computational analyses to understand biodynamic excisional skin tension lines. Sci Rep (2016) 6:30117. 10.1038/srep30117 27453542 PMC4958993

[5] Paul SP Golden spirals and scalp whorls: nature’s own design for rapid expansion. PLoS One (2016) 11(9):e0162026 10.1371/journal.pone.0162026 27583520 PMC5008782

[6] Danielson DA Wrinkling of the human skin. J Biomech (1977) 10(3):201–4. 10.1016/0021-9290(77)90059-8 858725

[7] Zollner AM Buganza Tepole A Gosain AK Kuhl E. Growing skin: tissue expansion in pediatric forehead reconstruction. Biomech Model Mechanobiol (2012) 11(6):855–67. 10.1007/s10237-011-0357-4 22052000 PMC3425448

[8] Buganza Tepole A Gosain AK Kuhl E Stretching skin: the physiological limit and beyond. Int J Non Linear Mech (2011) 47(8):938–49. 10.1016/j.ijnonlinmec.2011.07.006 23459410 PMC3583021

[9] Rodriguez EK Hoger A McCulloch AD. Stress-dependent finite growth in soft elastic tissues. J Biomech (1994) 27:455–67. 10.1016/0021-9290(94)90021-3 8188726

[10] Garikipati K The kinematics of biological growth. Appl Mech Rev (2009) 62(3):030801 10.1115/1.3090829

[11] Costa DJ Walen S Varvares M Walker RJ. Scalp rotation flap for reconstruction of complex soft tissue defects. J Neurol Surg B Skull Base (2016) 1:32–7. 10.1055/s-0035-1556874 26949586 PMC4777623

[12] Paul SP Norman RA Rotation Flaps of the Scalp: Study of the Design, Planning and Biomechanics of Single, Double and Triple Pedicle Flaps. Clinical Cases in Skin Cancer Surgery and Treatment. Switzerland: Springer International Publishing (2016).

[13] Larrabee WF Sutton D. The biomechanics of advancement and rotation flaps. Laryngoscope (1981) 91(5):726–44. 10.1288/00005537-198105000-00005 7231022

[14] Wunderlich RC Heerema NA Hair crown patterns of human newborns. Studies on parietal hair whorl locations and their directions. Clin Pediatr (1975) 14(11):1045–9. 10.1177/000992287501401111 1183129

[15] Samlaska CP James WD Sperling LC. Scalp whorls. J Am Acad Dermatol (1989) 21(3):553. 10.1016/S0190-9622(89)70225-5 2674215

[16] Emmett AJ The closure of defects by using adjacent triangular flaps with subcutaneous pedicles. Plast Reconstr Surg (1977) 59:45–52. 318753 10.1097/00006534-197701000-00008

[17] Anderson DB Kauffman RG Kastenschmidt LL. Lipogenic enzyme activities and cellularity of porcine adipose tissue from various anatomical locations. J Lipid Res (1972) 13(5):593–9. 10.2307/2037865 5075506

[18] Mukhopadhyay U Logarithmic spiral – a splendid curve. Resonance (2004) 9(11):39 10.1007/BF02834971

[19] Hakim V Karma A Spiral wave meander in excitable media: the large core limit. Phys Rev Lett (1997) 79(4):665 10.1103/PhysRevLett.79.665

[20] Chen J-X Guo M-M Ma J Termination of pinned spirals by local stimuli. EPL (2016) 113:3 10.1209/0295-5075/113/38004

